# Deletion of Miro1 in airway club cells potentiates allergic asthma phenotypes

**DOI:** 10.3389/falgy.2023.1187945

**Published:** 2023-06-12

**Authors:** Sierra Bruno, Amelia Lamberty, Margaret McCoy, Zoe Mark, Nirav Daphtary, Minara Aliyeva, Kelly Butnor, Matthew E. Poynter, Vikas Anathy, Brian Cunniff

**Affiliations:** ^1^ Department of Pathology and Laboratory Medicine, University of Vermont, Burlington, VT, United States; ^2^ Department of Medicine, University of Vermont, Burlington, VT, United States

**Keywords:** *Miro1*, asthma, inflammation, mitochondria, house dust mite (HDM)

## Abstract

Mitochondria are multifaceted organelles necessary for numerous cellular signaling and regulatory processes. Mitochondria are dynamic organelles, trafficked and anchored to subcellular sites depending upon the cellular and tissue requirements. Precise localization of mitochondria to apical and basolateral membranes in lung epithelial cells is important for key mitochondrial processes. Miro1 is an outer mitochondrial membrane GTPase that associates with adapter proteins and microtubule motors to promote intracellular movement of mitochondria. We show that deletion of *Miro1* in lung epithelial cells leads to perinuclear clustering of mitochondria. However, the role of Miro1 in epithelial cell response to allergic insults remains unknown. We generated a conditional mouse model to delete Miro1 in Club Cell Secretory Protein (CCSP) positive lung epithelial cells to examine the potential roles of Miro1 and mitochondrial trafficking in the lung epithelial response to the allergen, house dust mite (HDM). Our data show that Miro1 suppresses epithelial induction and maintenance of the inflammatory response to allergen, as Miro1 deletion modestly induces increases in pro-inflammatory signaling, specifically IL-6, IL-33, CCL20 and eotaxin levels, tissue reorganization, and airway hyperresponsiveness. Furthermore, loss of Miro1 in CCSP^+^ lung epithelial cells blocks resolution of the asthmatic insult. This study further demonstrates the important contribution of mitochondrial dynamic processes to the airway epithelial allergen response and the pathophysiology of allergic asthma.

## Introduction

Allergic asthma is a complex, chronic inflammatory condition of the small airways that affects as many as 300 million people of all ages worldwide, including 25 million Americans (about 8.5% of the United States' population) (1–3). Generally, a combination of environmental and genetic factors can cause allergic asthma (4, 5) however, allergen exposure is the single most important cause. The main phenotypic drivers of acute episodes, or “attacks,” of airway inflammation are increased mucus production, and obstruction and narrowing of the airways (6). These disruptive changes to the airway epithelium, the first line of protection against external insults and pathogens, can lead to some of the classical symptoms associated with allergic asthma such as chest tightness, difficulty breathing, wheezing, and persistent coughing (7). In allergic asthma, the airway epithelium is frequently injured by allergens, leading to the secretion of pro-inflammatory cytokines and chemokines, as well as the recruitment and activation of innate and adaptive immune cells (8). Chronic activation of these pro-inflammatory molecules potentiates structural changes in the airways such as thickened basement membranes, shedding of the surface epithelium, subepithelial fibrosis, mucus hyperplasia and metaplasia, and increased angiogenesis surrounding the airways (9). These structural changes to the airways lead to functional changes, contributing to symptoms including difficulty breathing.

Currently, there is no cure for allergic asthma. Avoidance of triggers and the use of bronchodilators and anti-inflammatory drugs such as inhaled rapid-acting β-2 adrenergic agonists and oral corticosteroids are commonly recommended for the management of symptoms. However, these current treatment options, and even contemporary biological therapeutics, fail in upwards of 10% of patients or have serious side effects (10, 11), making it necessary to study novel molecular targets to better understand the pathophysiological nature of allergic asthma and to explore alternative therapeutic options.

Ample evidence suggests that mitochondria play a key role in lung health and pathophysiology (12–15). Mitochondria, the “powerhouses of the cell,” are the main producers of adenosine triphosphate (ATP). Moreover, mitochondria are a primary source of reactive oxygen species (ROS) (16), and can actively buffer calcium (Ca^2+^) (17). Following cellular stress, mitochondrial membranes can be damaged and may become dysfunctional. Mitochondrial dysfunction in respiratory diseases is characterized by ROS accumulation, loss of membrane potential, mitochondrial Ca^2+^ overload, mitochondrial DNA mutation or release, and mitophagy dysregulation (18). Increased production of ROS has been implicated in the development of chronic inflammatory lung conditions such as asthma (11, 19). As a result, these double-membraned organelles change in size, shape, and distribution depending on intracellular mitochondrial metabolite demands (20–22). Our previous work showed that mitochondrial fission is an early event following exposure of bronchial epithelial cells to house dust mite and that knockout of the pro-fission protein Dynamin Related Protein 1 (DRP1) exacerbates asthmatic phenotypes (23). Within the cell, mitochondria are transported on the microtubule-associated molecular motors kinesin and dynein (24–26), and tethered to these motors by a protein complex composed of trafficking kinesin protein 1 and 2 (TRAK1/2) and Mitochondrial Rho GTPase 1 and 2 (Miro1/2) (27–32). Miro1 shares approximately 60% homology with Miro2, but evidence shows Miro1 is the primary adaptor protein required for the subcellular positioning of mitochondria within differentiated cells (33, 34). The dynamic reorganization of mitochondrial networks is important in highly active cells, such as airway epithelial cells, due to their increased need in energy to maintain the functionality of the airways. Miro1 is critical for proper subcellular positioning of mitochondria in fibroblasts and distribution of mitochondrial derived molecules including ROS (35) and ATP (36). Evidence suggests that mitochondria are positioned at the apical and basolateral poles of polarized airway epithelial cells to constrict calcium signaling (37), and provide energy for mucus to be secreted and cleared (38–41), which becomes critical during high energy demand events such as stress or injury. Recent *in vitro* and *in vivo* models of COPD suggest that Miro1 plays a contributing role to this pathology (42, 43). Miro1 has been implicated in mesenchymal stem cell transfer of mitochondria to damaged lung epithelia following inhibition of the electron transport chain and allows for suppression of the allergic asthma phenotype in mouse models of the disease (17). Until now, the contribution of Miro1 in the context of allergic asthma remained unexplored.

Herein, we developed a novel mouse model to study Miro1 biology in the context of allergic asthma, in which we hypothesize that epithelial deletion of Miro1 leads to exacerbated inflammatory responses following chronic allergen exposure. We find that epithelial ablation of Miro1 in club cell secretory protein (CCSP) positive lung epithelial cells followed by exposure to the complex allergen house dust mite (HDM) leads to significant changes in tissue architecture and organization, inflammatory cell infiltrates, airway hyperresponsiveness, and forced pressure volume. Loss of Miro1 also disrupts the resolution phase following allergen exposure, providing evidence for the importance of Miro1 in mediating allergen-induced responses in lung epithelial cells.

## Materials and methods

### Study approval

All mouse studies were approved by the Institutional Animal Care and Use Committee of the University of Vermont, Burlington, VT, USA under protocol number PROTO202000216.

### House dust Mite

HDM (XPB70D3A2.5, Stallergenes Greer, Lenoir, NC, USA) was suspended in Phosphate Buffered Saline (PBS). HDM concentration was determined by total protein concentration in HDM vial.

### Epithelial tissue-specific transgenic mice

To achieve conditional airway epithelial specific deletion, C57BL/6 mice containing floxed Miro1 alleles (Miro1^flx/flx^) (34) were crossed with double transgenic mice containing a Club Cell secretory protein (CCSP) promoter fused to a reverse tetracycline trans activator (rTetA) and a Tet operon fused to Cre recombinase (TetOP-Cre), termed CCSP-rTetA/TetOP-Cre (44), to generate triple transgenic (CCSP-rTetA/TetOP-Cre/Miro1^flx/flx^) mice, referred to as *ΔEpi-Miro1*. Mice containing the three gene inserts were used to conditionally delete Miro1 in CCSP positive airway epithelial cells upon exposure to doxycycline-containing food (6 g/kg; Purina Diet Tech, St Louis, Mo) for **7** days. Littermates missing one of the three gene inserts (CCSP-rtTA, TetOP-Cre, or Miro1^flx/flx^) also on the doxycycline-containing food were used as control mice. Both female and male mice were used in this study and distributed equally among groups.

### Mouse models of allergic asthma

An established model of allergic asthma was used for all experiments (45, 46). In this model, 25 µg of HDM or PBS was administered to anesthetized control and *ΔEpi-Miro1* mice via nasopharyngeal aspiration. Mice were sensitized to the allergen 7 days after the start of the doxycycline-containing food. In the single model of allergen challenge, mice are sensitized on days 0 and 7 and challenged on day 14 with harvest 4 h later. In the multiple challenge model, the allergic response in mice was boosted 7 days after sensitization and challenged for five consecutive days 14 days after sensitization with HDM or PBS. Mice were studied 24 h following the last allergen challenge.

### Airway hyperresponsiveness assessment

Mice were anesthetized using sodium pentobarbital (90 mg/kg) via intraperitoneal injection and tracheotomized using 18-guage cannulas. Mice were mechanically ventilated at a rate of 200 breaths/min using FlexiVent computer controlled small-animal ventilator (SCRIREQ, Montreal, QC, Canada). Airway hyperresponsiveness parameters including Newtonian resistance (Rn), tissue dampening (G), and tissue elastance (H) were measured in the mice after exposure to increasing concentrations (12.5 mg/ml, 25 mg/ml, and 50 mg/ml) of aerosolized methacholine. Measurement of lung mechanics presented are the average of three measurements encompassing the peak response.

### Bronchoalveolar lavage fluid collection and processing

Bronchoalveolar lavage fluid (BALF) was collected by washing the airways with 1 ml of sterile PBS. Cells were isolated via centrifugation and total cell counts were determined using a hemocytometer (3110, Hausser Scientific, Horsham, PA, USA). Cytopsins were conducted and cells were stained using Hema3 stain reagents (Fisher Scientific, Waltham, MA, USA) to obtain differential cell counts from a minimum of 300 cells.

### Tissue processing

Following euthanasia, left lung lobe tissue was collected, inflated, and fixed in 4% paraformaldehyde for 24–48 h at room temperature. Formaldehyde-fixed lung tissue was sent to the University of Vermont Medical Center for paraffin embedding. Tissue blocks were serially sectioned at a 5 μm thickness using a Leica 2030 manual paraffin microtome (Microscopy Imaging Center, University of Vermont). Tissue sections were mounted onto glass slides and dried in an oven at 52°C for 60 min. De-paraffinization and tissue rehydration for all staining procedures was achieved by immersing the glass slides through the following solutions: three 15 min xylene washes, two 5 min washes of 100% ethanol and 95% ethanol, and a 5 min wash of 70% ethanol, 50% ethanol, and dH_2_O.

### *Miro1* immunohistochemistry

Lung antigen retrieval was achieved by submersing slides in a DAKO antigen retrieval solution for 20 min at 95°C, slides were then allowed to cool down for 20 min at room temperature and rinsed in three 5 min PBS washes. Lung tissue slides were blocked in a 10% H_2_O_2_ in methanol solution for 15 min, followed by seven 5 min PBS washes. A 2.5% normal goat serum protein block (Vector Laboratories) was put on the slides for 15 min, followed by overnight incubation of primary α-Miro1 antibody (ABIN635090) diluted to a 1:200 concentration in PBS at 4°C. Following overnight incubation, slides were rinsed in seven 5 min PBS washes and a ImmPRESS polymer reagent (Vector Laboratories) was put on the tissue for 30 min at room temperature. Tissue was rinsed in seven 5 min PBS washes and exposed to a diaminobenzidine (DAB) peroxidase solution (Vector Laboratories) for immunohistochemical staining. Tissue was rinsed in dH_2_O and counterstained with hematoxylin and ammonium hydroxide. Images were captured at 20× magnification by Leica VERSA8 whole slide imager (Microscopy Imaging Center, University of Vermont).

### Fluorescence staining and imaging

Antigen retrieval was done by heating slides for 20 min at 95°C in sodium citrate buffer with 0.05% TWEEN-20 then rinsed in dH_2_O. Sections were then blocked for 1 h in 1% BSA in PBS, followed by incubation using the following primary antibodies: TOMM20 (Millipore, MABT166), at 1:250 and CC10 (Santa Cruz Biotechnology, sc-390313), at 1:250; overnight at 4°C. Slides were then washed 3 × 5 min in PBS and incubated with species-specific Alexafluor-488- or Alexafluor-647-conjugated secondary antibodies, and counterstained with DAPI in PBS at 1:4,000 for nuclear localization. Sections were imaged using a Nikon A1R Confocal laser-scanning microscope. Images were captured at 20× and/or 40× magnification. The image files were converted to Tiff format. Brightness and contrast were adjusted equally in all images. The distance from the closest mitochondria to the apical membrane was measured using multiple line scans/cell and averaged using ImageJ.

### Mucus metaplasia quantification

Periodic Acid Schiff (PAS) staining was conducted to assess mucus metaplasia. Lung tissue slides were immersed in 0.5% periodic acid for 10 min, followed by three 5 min rinses in dH_2_O, and 30 min in Schiff reagent. Next, two 1 min washes in 0.55% potassium metabisulfite, followed by a 10 min rinse under running water. Tissue was counterstained by immersing slides for 10 min in hematoxylin, rinsed under running water for 5 min, and 2 dips in 0.5% lithium carbonate. Images were captured at 20× magnification by Leica VERSA8 whole slide imager (Microscopy Imaging Center, University of Vermont). Mucus metaplasia was measured in the airways by quantitating the positively PAS-stained area using the Positive Pixel Count algorithm of Leica Aperio ImageScope Software (Aperio Technologies, San Diego, CA, USA). The Positive Pixel Count algorithm outputs were used to determine the positive PAS-stained area number of strong positive pixels normalized to lung tissue area.

### Remodeling quantification

Masson's trichrome staining was conducted to assess remodeling changes. Lung tissue slides were immersed for 1 h in Bouin's solution at 56°C and cooled to room temperature. Tissue was rinsed under running water until stain disappeared. Tissue was stained for 10 min in Wiegert hematoxylin and washed under running water for 10 min. Next, tissue was stained for 2 min in Biebrich Scarlet-Acid Fuchsin solution, followed by a rinse in dH_2_O, and then immersed for 10–15 min in a phosphomolybdic/phosphotungstic acid solution. The last stain was 5 min in aniline blue, followed by a rinse in dH_2_O, and 3–5 min in a 1% acetic acid solution. Nuclei were stained in black; cytoplasm, keratin, and muscle fibers were stained in red; and collagen was stained in blue. Images were captured at 20× magnification by Leica VERSA8 whole slide imager (Microscopy Imaging Center, University of Vermont). Representative images of the small airways were captured for each experimental group. Images were de-identified and blindly scored using an arbitrary unit (A.U.) scale ranging from 1 to 8 by ∼6 separate individuals. The arbitrary unit scoring scale assessed changes in remodeling including collagen deposition, immune cell infiltration, epithelial layer thickening, and alveolar space changes. Lower scores signified little to no remodeling changes and higher scores signified increased remodeling changes. Mean A.U. scores per evaluator were obtained and these were averaged per experimental condition.

### ELISAs

Right side lung lobes were flash frozen immediately after harvest and crushed to make lysates in buffer containing 137 mM Tris HCl (pH 8.0), 130 mM NaCl, and 1% NP-40. Samples were normalized to total lung protein and used to assess the abundance of IL-6, IL-33, CCL20, Eotaxin-1 (DuoSet ELISA Kits, R&D Systems, Minneapolis, MN, USA), IL-4, IL-13 (eBioscience Kits, Thermo Fisher Scientific, Waltham, MA, USA), and MUC5AC (Novus Biologicals, Littleton, CO, USA) per manufacturer's instructions.

### Statistical analyses

Normal data were analyzed by Students t-test, one-way ANOVA and Tukey's multiple comparisons post-test using GraphPad Prism. A *p*-value <0.05 was considered significant. Data were averaged and expressed as the mean ± SEM.

## Results

### Epithelial *Miro1* deletion restricts mitochondria to the basolateral side of the cell

**Figure 1:**
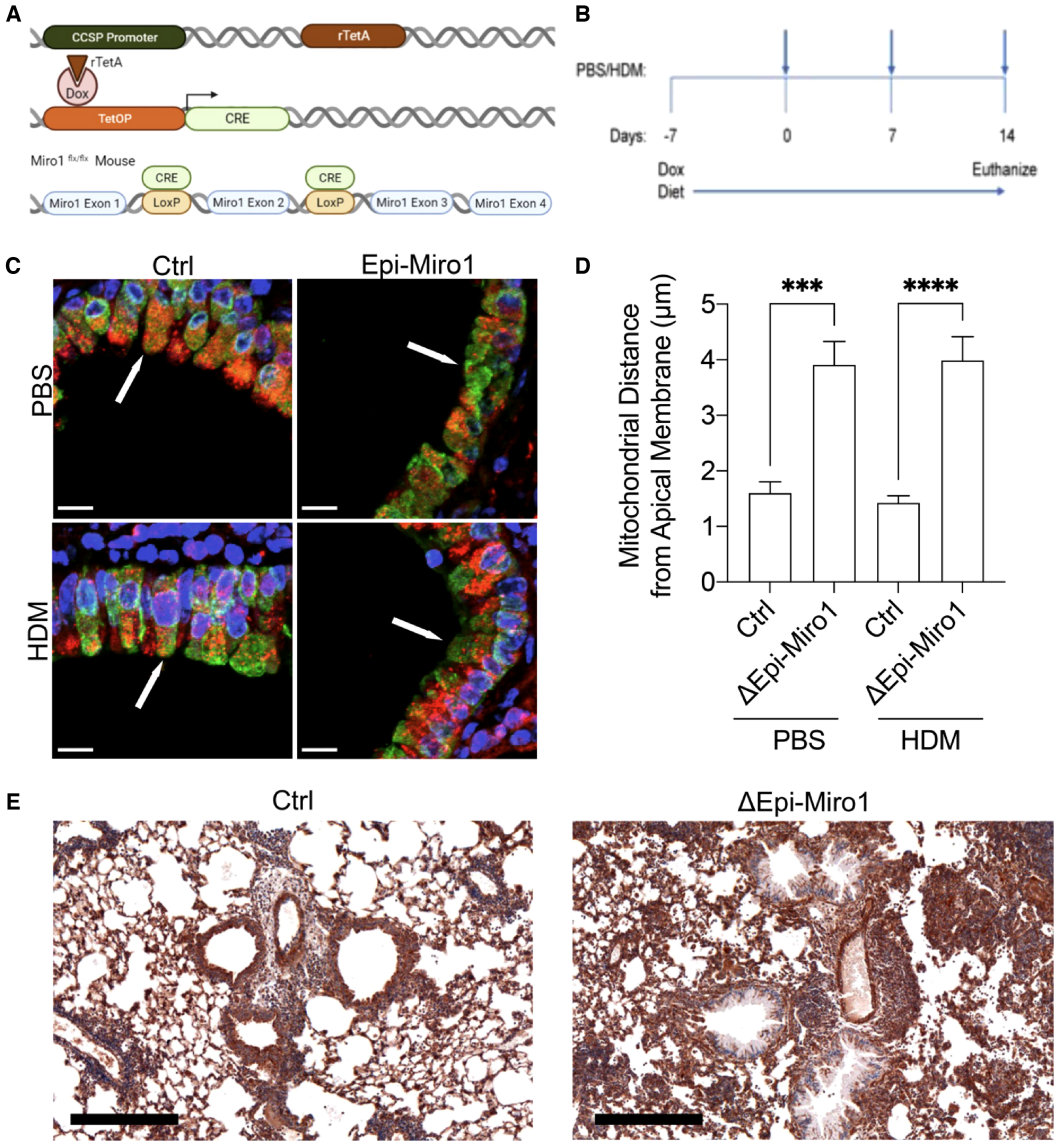
Miro1 deletion from club cells constricts mitochondria to the basolateral side of the cell. (**A**) Schematic of the doxycycline inducible deletion of Miro1 gene from CCSP positive epithelial cells. (**B**) Protocol schematic for the deletion of Miro1 via doxycycline containing food and single challenge HDM exposure protocol. Mice are sensitized on days 0 and 7 and challenged on day 14 with harvest 4 hours later. (**C**) Representative immunofluorescent images of TOMM20 (Red), CC10 (Green) and DAPI (Blue) in mouse lung tissue from representative mice. Arrows indicate areas of mitochondrial density in Ctrl mice that are lost at apical membranes in Epi-Miro1 mice. Scale Bar = 10 μm (**D**) Quantification of mitochondrial distance from the apical surface of club cells, *n* = 8–10 cells/airway from 5 mice per group; Two-way ANOVA with 2-stage linear set-up procedure. ***p < .001 *vs*. corresponding Ctrl group. Error bars represent mean ± SEM. (**E**) Immunohistochemical images of Miro1 expression in Ctrl and ΔEpi-Miro1 mice. Note loss of Miro1 IHC expression in the epithelial cell layer of ΔEpi-Miro1 mice. Scale bar = 300 μm

To begin to understand the impact of Miro1 mediated mitochondrial trafficking on epithelial response to complex allergens, we generated an *in vivo* model system to conditionally delete *Miro1* from CCSP + club cells in the mouse airway epithelium through the administration of doxycycline (Figure 1A). The CCSP-Cre mouse has been shown to restrict Cre expression to the lung (47, 48), although some studies indicate a possible subset of cells where CCSP is expressed during development (49) or in models of infection (50). Doxycycline-containing diet was provided to mice for 7 days prior to initiation of house dust mite (HDM) exposure to allow effective deletion. This diet was maintained throughout the duration of the dosing protocol until euthanasia. In an acute allergen recall challenge model, mice were sensitized on days 0 and 7 and challenged on day 14 with either 25 µg of HDM or phosphate buffered saline (PBS) control via intranasal inhalation and were harvested 4 h after the challenge (Figure 1B). Alteration of mitochondrial trafficking in club cells following *Miro1* deletion were confirmed using immunofluorescence on fixed lung tissue sections with TOMM20 labeling the mitochondria and CC10 labeling club cells. Nuclei were stained with DAPI. *Miro1* deletion induced mitochondrial constriction to the basolateral side of airway club cells and away from the apical surface (Figures 1C,D). We did not evaluate the morphology of mitochondria under these conditions due to limitations in resolution. Loss of Miro1 expression in lung epithelial cells of *Miro1*-deleted mice at time of harvest was observed in tissues stained with anti-Miro1 antibody by immunohistochemistry (Figure 1E).

### Epithelial *Miro1* deletion does not alter initiation of inflammatory cell infiltration into the airways but does enhance pro-inflammatory cytokine abundance following single HDM challenge

**Figure 2:**
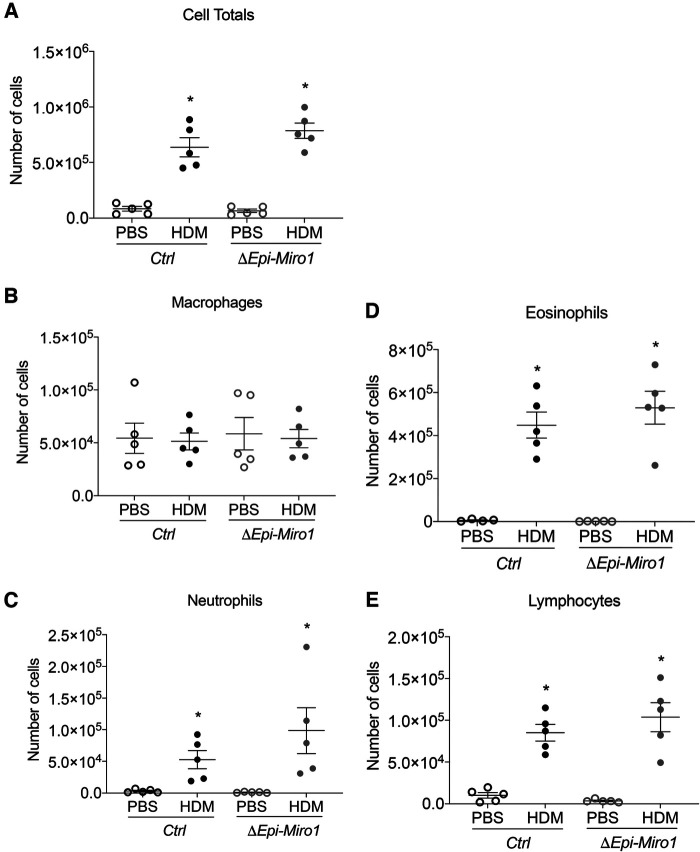
Epithelial Miro1 deletion does not significantly alter inflammatory cell recruitment following single HDM challenge. (**A**) Total cell infiltrates into the airways collected in the BALF, *n* = 5 mice per group: Two-way ANOVA with 2-stage linear set-up procedure, *p < 0.05 vs. corresponding PBS group. Error bars represent mean ± SEM. (**B–E**) Inflammatory cell-specific infiltrates present in the BALF, *n* = 5 mice per group; Two-way ANOVA followed by Sidak multiple comparisons test. *p < 0.05 vs. corresponding PBS group. Error bars represent mean ± SEM.

Bronchoalveolar lavage fluid (BALF) was collected and used to assess inflammatory cell infiltration into the airways of the mice following HDM exposure. Total immune cell counts present in the BALF was marginally, though not significantly, enhanced in *Miro1*-deleted (*ΔEpi-Miro1*) mice exposed to HDM when compared to control (*Ctrl*) HDM-exposed mice (Figure 2A). This trending increase is attributed to slight increases in the numbers of eosinophils, neutrophils, and lymphocytes, with no discernable change in overall macrophage abundance in the airways (Figures 2B–E).

**Figure 3:**
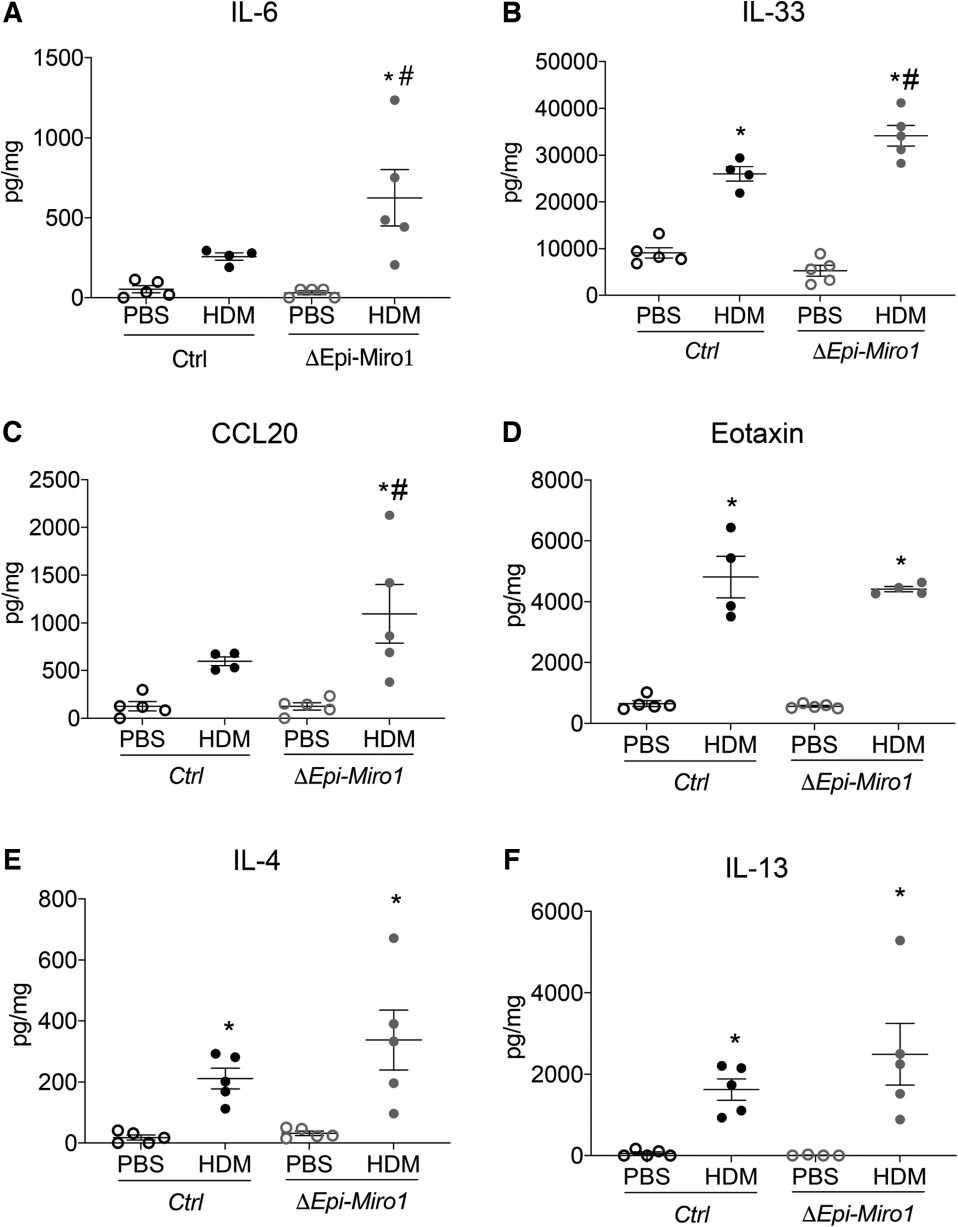
Epithelial Miro1 deletion enhances pro-inflammatory abundance following single HDM challenge. (**A,B**) ELISAs of pro-inflammatory cytokine abundance in lung tissue lysates, *n* = 5 mice per group; (**C,D**) ELISAs of pro-inflammatory chemokine abundance in lung tissue lysates, *n* = 5 mice per group; (**E,F**) ELISAs of Th2 cytokine abundance in lung tissue lysates, *n* = 5 mice per group: Two-way ANOVA followed by Sidak multiple comparisons test. *p < 0.05 vs. corresponding PBS group, #*p* < 0.05 vs. corresponding Ctrl group. Error bars represent mean ± SEM.

Though there were no significant cellular differences in the BALF between *Ctrl* and *ΔEpi-Miro1* HDM-exposed mice, there were significant enhancements in several pro-inflammatory cytokines and chemokines in the lung tissue of *Miro1* deleted HDM-exposed mice. More specifically, IL-6 and IL-33, two pro-inflammatory cytokines produced by airway epithelia known to play key roles in severity of asthma inflammation (20, 21), were modestly yet significantly upregulated in HDM-exposed mice following *Miro1* deletion in club cells when compared to *Ctrl* HDM-challenged mice (Figures 3A,B). Further, the chemokine CCL20, produced by airway epithelial cells and known to play a significant role in mucus metaplasia, eosinophil recruitment, and IgE production in response to allergen (22, 23), was also significantly upregulated in HDM-exposed *Miro1*-deleted mice compared to *Ctrls*. (Figure 3C). Eotaxin, another key pro-inflammatory chemokine for eosinophil recruitment, did not differ between groups (Figure 3D). There were slight but not significant increases in production of the Th2 cytokines IL-4 and IL-13 as well (Figures 3E,F). These data suggest that *Miro1* may help mediate the pro-inflammatory signaling response to allergen by airway epithelia.

### Epithelial *Miro1* deletion enhances lymphocyte infiltration and pro-inflammatory signaling following multiple HDM challenges

**Figure 4:**
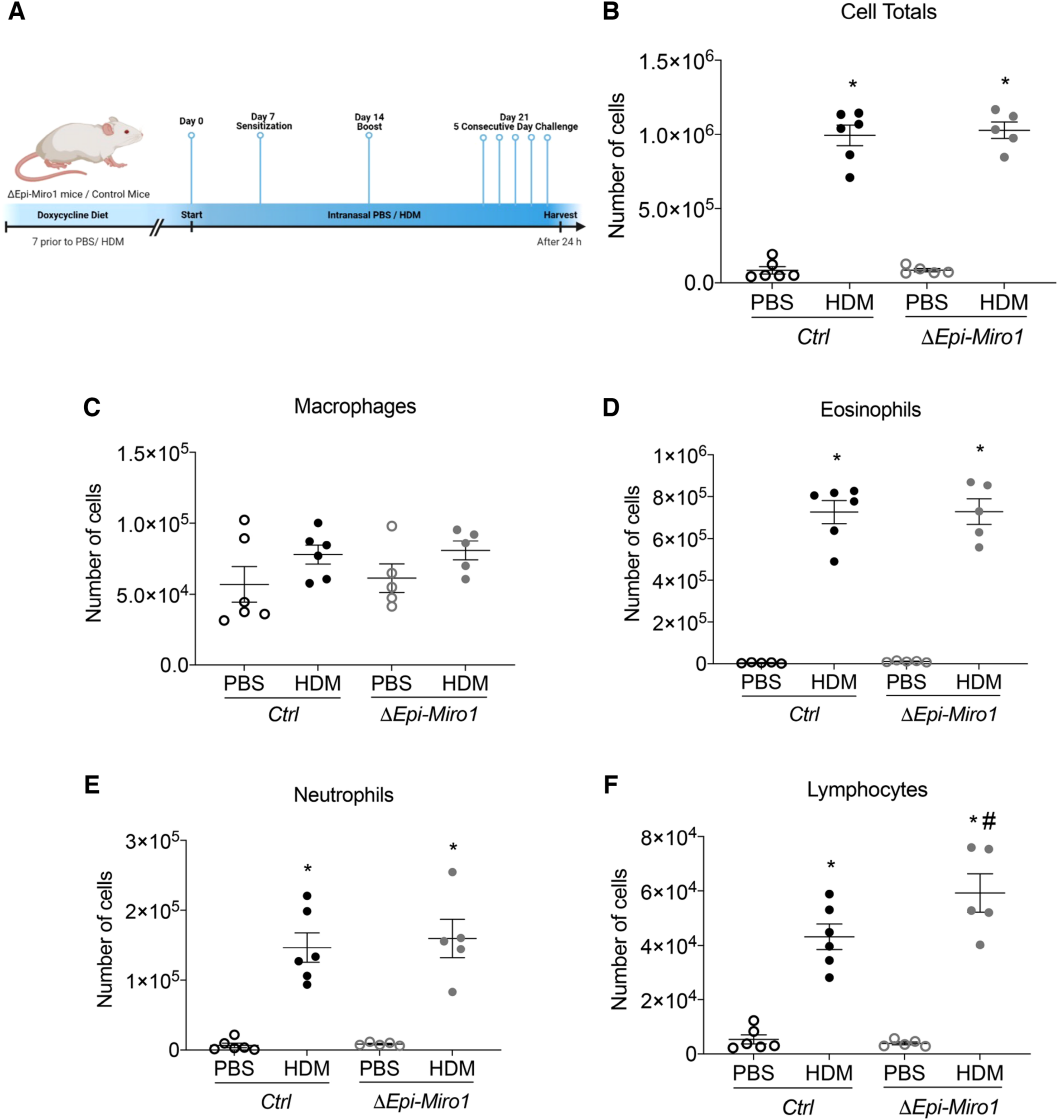
Epithelial Miro1 deletion significantly increases lymphocyte recruitment following multiple HDM challenges. (**A**) Schematic of deletion and multiple HDM challenge protocol; (**B**) Total cell infiltrates into the airways collected in the BALF, *n* = 5–6 mice per group: Two-way ANOVA with 2-stage linear set-up procedure. (**C–F**) Inflammatory cell-specific infiltrates present in the BALF, *n* = 5–6 mice per group: Two-way ANOVA followed by Sidak multiple comparisons test. *p < 0.05 vs. corresponding PBS group, #*p* < 0.05 vs. corresponding Ctrl group. Error bars represent mean ± SEM.

We next aimed to determine whether these differences in initiation would translate to differences in progression and amplification of the inflammatory phenotype seen in a multi-challenge model of allergic asthma. We therefore utilized a challenge protocol in which we gave mice doxycycline 7 days before HDM sensitization. Mice where sensitized with 25μg HDM or PBS on days 0 and 7. Challenges occurred once a day on days 14 through 18, and mice were euthanized 24 h following the final challenge, on day 19. Mice were maintained on doxycycline-containing diet for the full duration of the experiment (Figure 4A). As with the single challenge protocol, there were no significant differences in total inflammatory cell infiltration into the BALF when comparing *Ctrl* and *ΔEpi-Miro1* HDM-challenged mice (Figure 4B). Quantitation of inflammatory cell populations that enter the airspace following allergen exposure revealed no significant differences in macrophages, eosinophils, or neutrophils (Figures 4C–E). However, a significant enhancement in the number of lymphocytes present in the airways of *Miro1*-deleted HDM-challenged mice was observed compared to *Ctrl* mice (Figure 4F).

**Figure 5:**
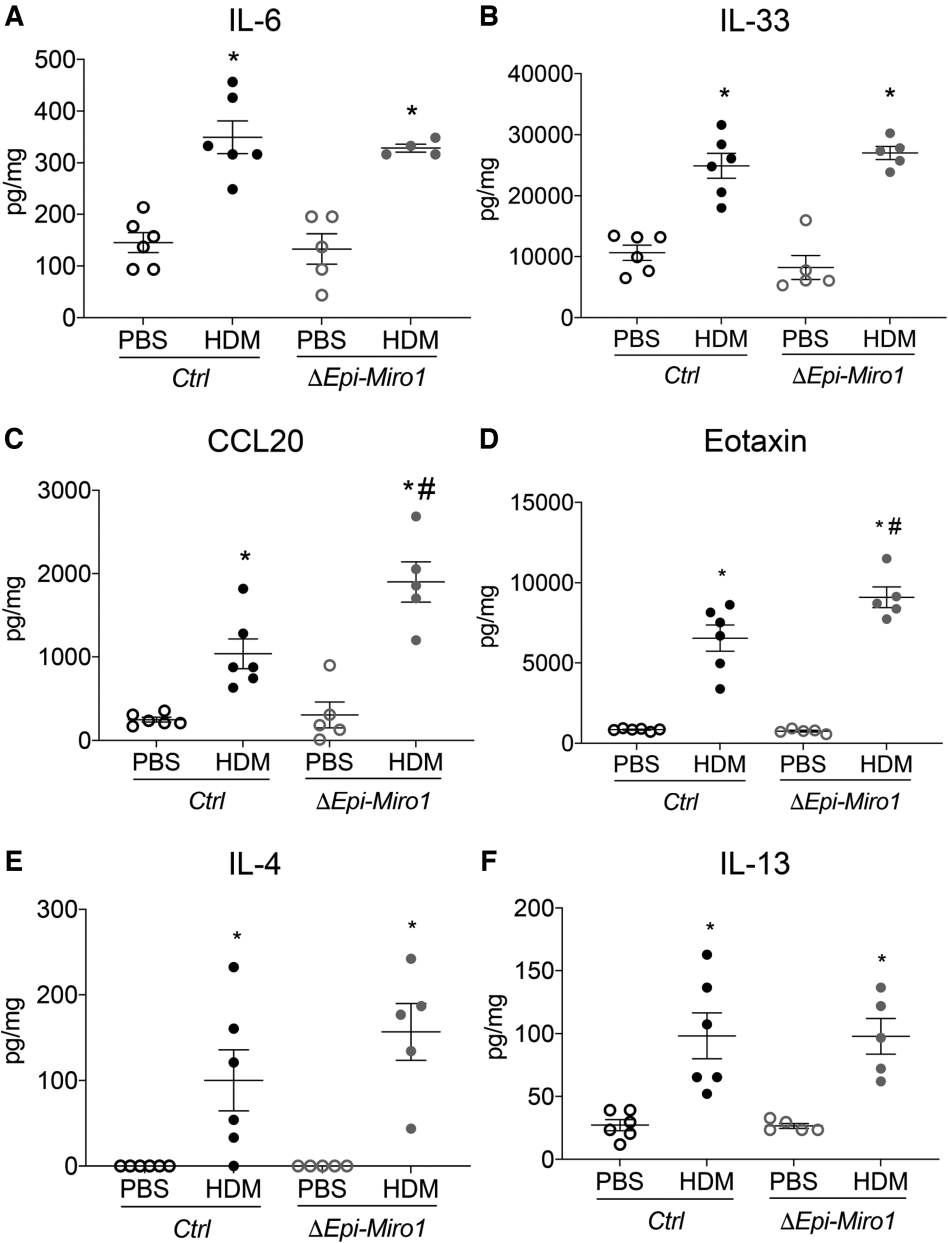
Epithelial Miro1 deletion significantly alters pro-inflammatory chemokine abundance following multiple HDM challenges. (**A,B)** ELISAs of pro-inflammatory cytokine abundance in lung tissue lysates, *n* = 5 mice per group; (**C,D**) ELISAs of pro-inflammatory chemokine abundance in lung tissue lysates, *n* = 5 mice per group; (**E,F**) ELISAs of Th2 cytokine abundance in lung tissue lysates, *n* = 5 mice per group; Two-way ANOVA followed by Sidak multiple comparisons test, *p < 0.05 vs. corresponding PBS group, #*p* < 0.05 vs. corresponding Ctrl group. Error bars represent mean ± SEM.

Pro-inflammatory cytokines and chemokines were also assessed, via ELISA, in lung tissue with this model. Though enhanced in the single challenge model of HDM exposure following *Miro1* deletion, there were no discernable differences in expression of pro-inflammatory cytokines IL-6 and IL-33 between *ΔEpi-Miro1* and *Ctrl* mice (Figures 5A,B). CCL20 and Eotaxin, however, were significantly enhanced in *Miro1* deleted mice challenged multiple times with HDM when compared to *Ctrl* HDM mice (Figures 5C,D). And, as with the single challenge protocol, *ΔEpi-Miro1* and *Ctrl* HDM mice had no significant differences in the abundance of Th2 cytokines IL-4 and IL-13, though *Miro1* deleted HDM-challenged mice did trend higher than *Ctrls* (Figures 5E,F). These data together demonstrate a modest enhancement of inflammation in a more protracted model of allergic asthma following *Miro1* epithelial deletion.

### Conditional deletion of *Miro1 in vivo* augments mucus metaplasia and tissue remodeling

**Figure 6:**
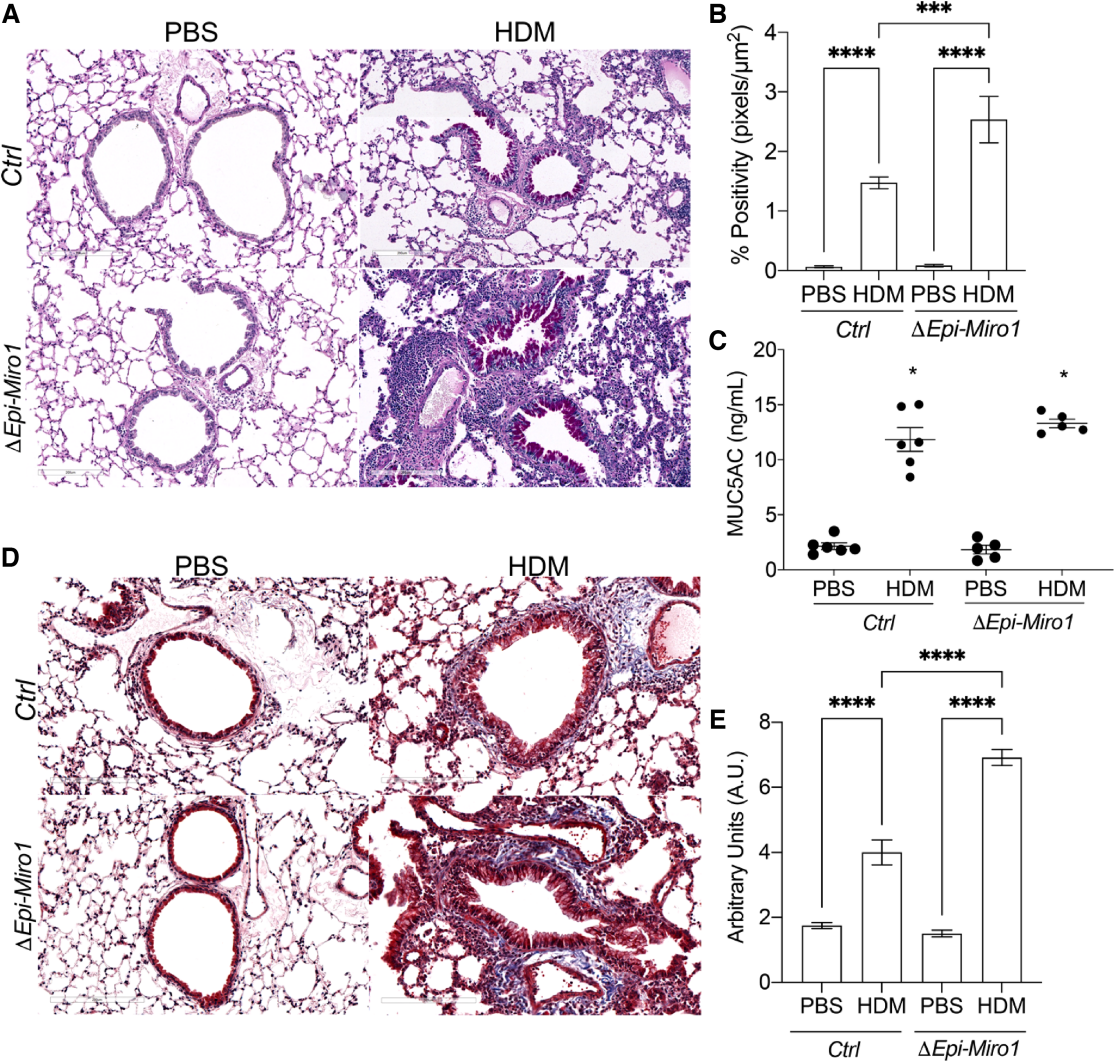
Conditional deletion of Miro1 *in vivo* augments mucus metaplasia and tissue remodeling (**A**) representative images of PAS staining in lung tissue sections from one experiment. (**B**) Quantification of PAS staining from multiple airways from *n* = 3–6 mice per group. (**C**) ELISA of MUC5AC in the BALF, *n* = 5–6 mice per group from one experiment: One-way ANOVA followed by Tukey multiple comparisons test. (**D**) Representative images of Masson's trichrome staining in lung tissue sections, *n* = 3–6. Scale bars are 200 µm. (**E**) Quantification of Masson's trichrome staining. The arbitrary unit scoring scale assessed changes in remodeling including collagen deposition, immune cell infiltration, epithelial layer thickening, and alveolar space changes. Lower scores signified little to no remodeling changes and higher scores signified increased remodeling changes. See materials and methods for scoring criteria. Error bars represent mean ± SEM. One-way ANOVA followed by Tukey multiple comparisons test, ***p = value < 0.001, ****p-value < 0.0001.

Mucus metaplasia is a hallmark of allergen-induced asthma and an indicator of its severity (51–53). Thus, we examined mucus metaplasia in lung tissue and MUC5AC abundance in the BALF after Miro1 deletion from the airway epithelium and HDM challenge. To assess mucus levels histologically, lung tissue was stained using the Periodic acid-Schiff stain (PAS) stain. Mucus staining was significantly increased in lung tissue of *ΔEpi-Miro1* mice challenged with HDM, which exhibited extensive goblet cell hyperplasia when compared to the HDM-challenged *Ctrls* (Figures 6A,B). PAS staining was strong and concentrated in the epithelial cell layer in *ΔEpi-Miro1* mice challenged with HDM (Figure 6A), suggesting epithelial Miro1 may regulate mucin secretion, further suppressing severity of the allergic response to HDM. Extensive immune cells were also observed in the surrounding lung tissue of *ΔEpi-Miro1* mice challenged with HDM (Figure 6A). There were no statistically significant differences in the MUC5AC levels in the BALF between *ΔEpi-Miro1* mice and *Ctrls* exposed to HDM in the BALF (Figure 6C), possibly due to cellular retention of mucus.

Next, we utilized Masson's trichrome (MT) staining to assess airway tissue remodeling, including collagen deposition, immune cell infiltration, epithelial hyperplasia, and alveolar space size in the lung tissue (Figure 6D). There were marked remodeling changes observed in the lung tissue of *ΔEpi-Miro1* mice challenged with HDM when compared to control mice challenged with HDM (Figure 6D). *ΔEpi-Miro1* exhibited more marked chronic small airway inflammation and reactive airway epithelial changes with more extensive peribronchiolar fibrosis (Figures 6D,E). These data suggest that Miro1 might help prevent severe remodeling changes in the airway epithelium following allergen exposure.

### Conditional deletion of *Miro1 in vivo* enhances airway hyperresponsiveness following methacholine challenge

**Figure 7:**
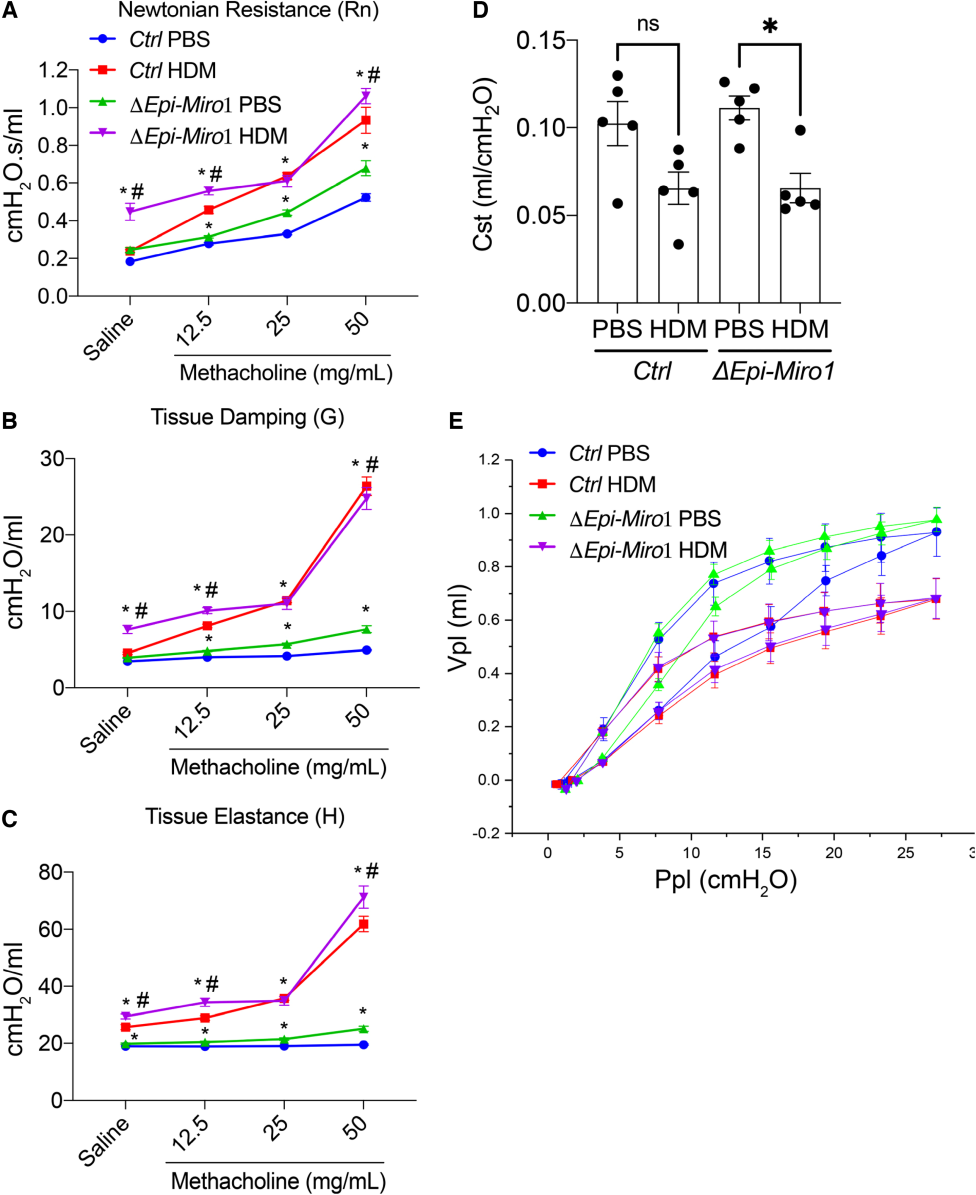
Epithelial Miro1 deletion significantly enhances AHR and cell death following multiple HDM challenges. (**A**) Newtonian Resistance (Rn) (**B**) Tissue Damping (G) and (**C**) Tissue Elastance (H) of control (Ctrl) and Epi-Miro1 mice challenged with PBS or HDM, *n* = 4–6 mice per group: Two-way ANOVA with 2-stage linear set-up procedure, *p < 0.05 vs. corresponding PBS group, #*p* < 0.05 vs. corresponding Ctrl group. Error bars represent mean ± SEM. (**D**) Static compliance (Cst) measurement in mice challenged with PBS or HDM, *n* = 4–6 mice per group: One-way ANOVA followed by Tukey multiple comparisons test, *p < 0.05. (**E**) Pressure volume curve analysis of indicated groups and treatments, *n* = 4–6 mice per group.

Methacholine-induced airway hyperresponsiveness (AHR) in the protracted HDM-induced allergic airway disease model was assessed. *ΔEpi-Miro1* mice exposed to both PBS and HDM showed slightly increased, but significant, Newtonian resistance, tissue dampening, and tissue elastance compared to wild-type control animals (Figures 7A–C). These alterations in the AHR parameters, together with an increase in inflammation and mucus abundance are suggestive of increased airway constriction and collapse following exposure to HDM in airway epithelial *Miro1*-deleted mice. We also measured static compliance (Cst), which reflects the elastic properties of the respiratory system at rest. Cst was reduced in both *Ctrl* and *ΔEpi-Miro1* mice exposed to HDM but only reached statistical significance in the *ΔEpi-Miro1* group (Figure 7D). Lastly, we conducted a pressure-volume (Vpl and Ppl) curve analysis to calculate plateau pressure (Ppl) related to the total volume (Vpl) of methacholine delivered to the animals. HDM lowered both the Vpl and Ppl compared to PBS treated animals in both control and *ΔEpi-Miro1* mice (Figure 7E).

### Resolution of AHR, pressure volume and static compliance is delayed in *ΔEpi-Miro1*

**Figure 8:**
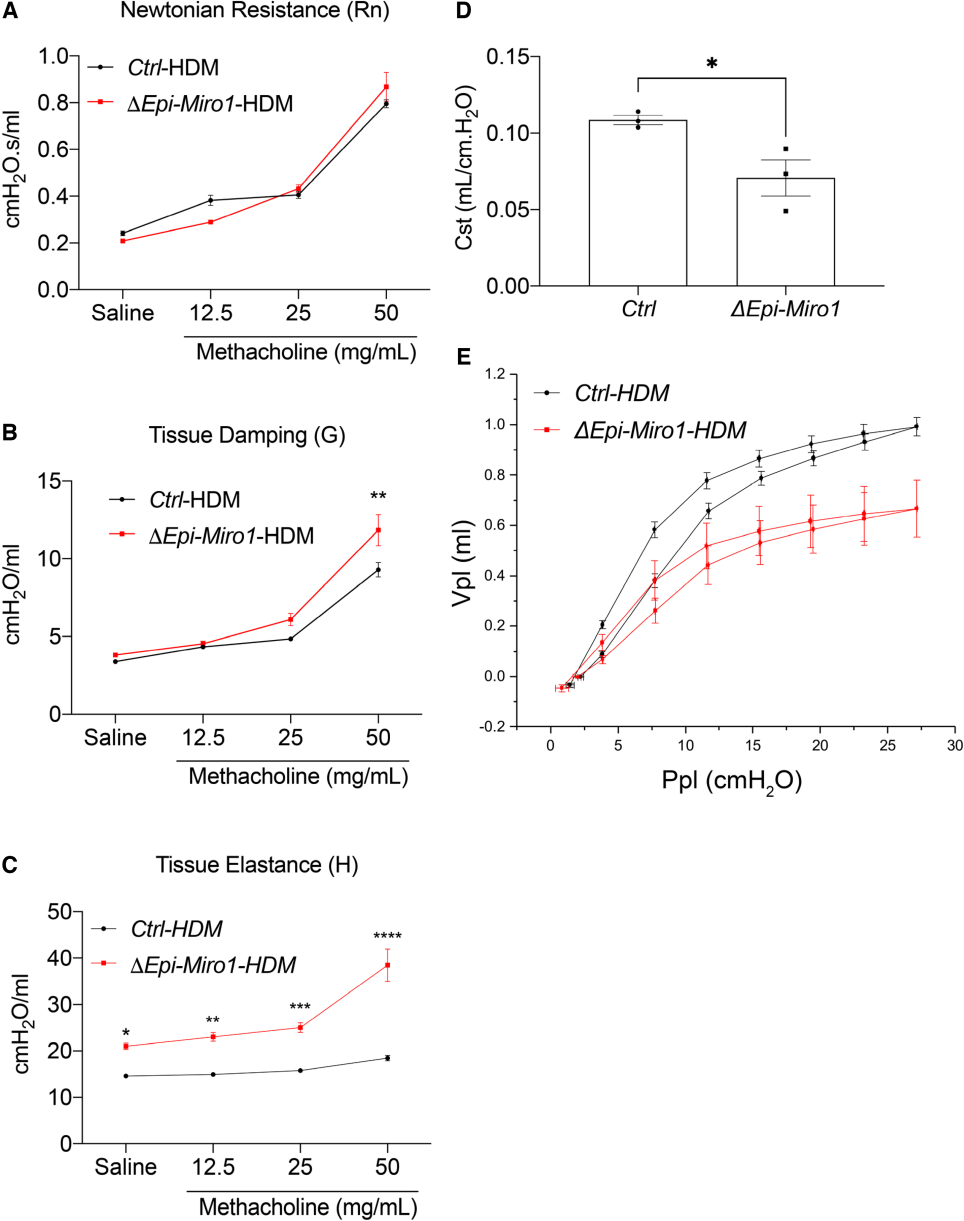
Resolution of AHR and pressure volume is delayed in epithelial Miro1 deleted mice compared to control mice. (**A**) Newtonian Resistance (Rn) (**B**) Tissue Damping (G) and (**C**) Tissue Elastance (H) of control (Ctrl) and Epi-Miro1 mice on the multiple challenge protocol and 2 weeks of no challenge, *n* = 3 mice per group, Two-way ANOVA with 2-stage linear set-up procedure. Error bars represent mean ± SEM. (**D**) Static compliance (Cst) measurement in mice challenged with PBS or HDM and allowed to resolve to 2 weeks, *n* = 3 mice per group, Students *t*-test, *p < 0.05. (**E**) Pressure volume curve analysis of indicated groups and treatments, *n* = 3 mice per group.

To evaluate resolution of the asthmatic phenotype induced by HDM exposure, we waited 2-weeks post HDM exposure in the multiple challenge protocol to evaluate cohorts of mice in *Ctrl* and *ΔEpi-Miro1* groups. AHR parameters of tissue damping and elastance were still significantly increased in *ΔEpi-Miro1* challenged compared to controls, whereas Newtonian resistance was comparable in both groups following methacholine inhalation (Figures 8A–C). Static compliance (Cst) measurements showed that Ctrl mice returned to control levels of approximately 0.10 ml/cmH_2_O while *ΔEpi-Miro1* mice maintained a significantly lower Cst (Figure 8D), comparable to animals harvested 24 h post HDM exposure (Figure 7D). Lastly, pressure-volume loop analysis showed resolution in Ctrl mice while *ΔEpi-Miro1* maintained a diminished pressure-volume loop (Figure 8E) comparable to animals harvested 24 h post-HDM (Figure 7E). Together these data indicate that deletion of Miro1 potentiates inflammatory responses and altered airway mechanics that fail to resolve following cessation of the HDM challenge.

## Discussion

The results from this study show that deletion of *Miro1* from club cells leads to an increased inflammatory cell influx and enhanced secretion of pro-inflammatory cytokines in lung tissue following chronic HDM exposure. *Miro1* epithelial deletion also leads to augmented mucus metaplasia and pronounced remodeling in the airways. Finally, *Miro1* deletion alters lung mechanics following chronic allergen insult. The role of Miro1-mediated mitochondrial trafficking upon exposure to a complex allergen in airway epithelial cells, and their associated inflammatory responses, had not been demonstrated in previous literature. Our results provide compelling evidence for the role of Miro1 in airway epithelial cells by mediating inflammatory responses associated with HDM exposure and build upon our previous studies showing the importance of mitochondrial DRP1 to this process (23). However, the mechanistic details underlying the observed phenotype remain unclear, including additional aspects of Miro1 biology (54, 55) that where next explored in this study.

Chronic inflammation of the lungs caused by aeroallergens such as HDM is a hallmark of the pathophysiology associated with allergen-induced asthma (56). In this study, *Miro1* was conditionally deleted from CCSP-positive airway epithelial cells in C57BL/6 mice. CCSP is predominantly expressed in club cells, a non-ciliated secretory epithelial cell subtype that is ubiquitously expressed in murine lungs (57, 58), although some studies indicate a possible subset of cells where CCSP is expressed during development (49) or in models of infection (50). *ΔEpi-Miro1* and *Ctrl* mice were sensitized, boosted, and exposed for 5 consecutive days in a chronic model of allergen-induced inflammation to determine the contribution of *Miro1* to HDM-induced lung inflammation. Our results show that epithelial deletion of *Miro1* leads to an inflammatory cell influx in the BALF, especially lymphocytes, as well as an increase of pro-inflammatory mediators including CCL20 and eotaxin in the whole tissue lysate following HDM exposure. It is possible that the increase of CCL20, a lymphocyte and dendritic cell chemoattractant, and eotaxin, an eosinophil chemoattractant, promotes the increase in inflammatory cells following *Miro1* deletion in airway epithelial cells. Specialized immunostainings on the lung tissue would be beneficial to determine the inflammatory cell profile in the observed sub-epithelial inflammation.

In agreement with our work, it was demonstrated that epithelial deletion of *Miro1* leads to heightened pro-inflammatory responses following exposure to cigarette smoke (43). However, the inflammatory response in allergen-induced asthma is characterized by eosinophilia associated with an increased number of T lymphocytes and mast cell activation. CD4+ T lymphocytes regulate chronic inflammation in asthma via the release of Th2 adaptive cytokines (59, 60), which can lead to the observed changes in airway hyperresponsiveness, mucus production, and airway remodeling. Moreover, *Miro1* plays a critical role in signaling for mitophagy and interacting with the Pink1/Parkin mitochondrial quality control system (61, 62), and compromised degradation of Parkin has been shown to promote inflammation and the release of mtDNA (63). However, mitophagy can activate apoptotic signaling pathways (64–66), an observation we made via the increase in the activity of caspases 3/7 (data not shown). Although we see non-significant increases in pro-inflammatory cytokines, Th2 cytokines, and inflammatory cell types *in situ*, future studies will measure these and other markers in the serum to assess their changes systemically. Our data suggest that *Miro1* regulates inflammatory responses, primarily lymphocyte recruitment, following HDM exposure.

Airway remodeling has long been considered an important feature of asthma that results from longstanding inflammation and can lead to airway hyperresponsiveness (67). Structural changes within the airway wall such as epithelial membrane thickening, hypertrophy of smooth muscle cells, and peribronchial fibrosis result in the pathology associated with asthma (68). In this study, *Miro1* deletion from epithelial cells led to prominent airway remodeling changes resulting in increased collagen deposition, heightened immune cell infiltration, and epithelial layer thickening, as demonstrated via Masson's trichrome staining. *Miro1* deletion also led to increased smooth muscle surrounding the perimetry of the airways, as shown through immunohistochemical staining for *α*-smooth muscle actin (data not shown). The observed increase in eotaxin in the whole tissue lysate could be attributed to the increase in airway smooth muscle following *Miro1* deletion, as airway smooth muscle has been shown to produce eotaxin and recruit eosinophils from systemic circulation (69). Moreover, other studies suggest that altered calcium homeostasis that leads to increased mitochondrial biogenesis results in increased bronchial smooth muscle mass (70). It is possible that lack of appropriate mitochondrial positioning in airway epithelial cells following *Miro1* deletion could lead to calcium level dysregulation and increased airway smooth muscle. Together with our inflammatory cell profiles, these data are suggestive that Miro1 helps attenuate inflammation-associated remodeling changes.

Mucus is primarily produced and secreted by goblet cells, a specific subset of airway epithelial cells (71, 72). Goblet cell hyperplasia and metaplasia have been associated with asthma severity (52, 73), with mucus plugs being the primary cause of death in asthma due to asphyxiation from intraluminal airway obstruction (53). Our data suggest that club cell specific deletion of *Miro1* leads to augmented mucus metaplasia as shown through the accumulation of mucus following exposure to HDM in the airway epithelium. However, there were no increases in mucin protein levels in the BALF after HDM exposure. Therefore, we hypothesize that mucins may remain in the secretory vesicles and not secreted into the intraluminal space following *Miro1* deletion. Studies have shown that ATP is required to initiate a signaling cascade that results in a Ca^2+^ triggered fusion of the mucin granules to the membranes (38–41). Recent literature examining the role of *Miro1* during alveolar formation observed a similar phenomenon where loss of epithelial *Miro1* compromised the release of platelet-derived growth factor (74). Mitochondrial positioning via Miro1 also supports subcellular gradients of ATP and ROS towards the cell periphery in fibroblasts (35, 36) that may be also be lost in lung epithelial cells following Miro1 deletion, contributing to disrupted exocytic release. We suspect that *Miro1* expression leading to the appropriate positioning of mitochondria in lung epithelial cells may be necessary for the secretion of mucins from airway epithelial cells, preventing more severe asthma phenotypes. However, it is unclear whether *Miro1* deletion from club cells changes the functionality of mucus producing cells if transdifferentiated, as murine asthma models have shown a dramatic shift in cell phenotypes in the epithelium resulting from club cell differentiation to mucus cells (75).

The functional consequence of asthma is reversible airflow limitation (76). Airway inflammation alone may cause airflow limitation or through inflammatory mediators that act directly on airway smooth muscle (77). Enhanced tissue remodeling and mucus hyperplasia have been associated with increased airway hyperresponsiveness (78, 79). Our data show that Miro1 deletion from epithelial cells leads to changes in airway mechanics independent of HDM exposure, suggesting intrinsic alterations within the epithelium. The observed changes in airway hyperresponsiveness in this study could be attributed to the heightened immune cell infiltration, mucus obstruction as a result of goblet cell metaplasia, and enhanced smooth muscle levels and remodeling changes. Of striking importance is the inability for asthmatic phenotypes to be resolved in *ΔEpi-Miro1* following a 2-week resolution phase. This information warrants investigation into Miro1 protein regulation in asthmatic models.

Results from this study suggest that Miro1-mediated mitochondrial trafficking contributes to the regulation of pro-inflammatory responses in the airway epithelium. Deletion of *Miro1* was shown to augment chronic HDM-induced inflammatory responses in the lungs, associated with pronounced inflammatory cell infiltration and remodeling changes in the mouse lungs leading to altered lung mechanics. These results indicate a possible role for *Miro1* in the development and progression of inflammatory responses and provide insights for the role of Miro1 in allergic airway diseases. Additional studies should be conducted to elucidate the mechanisms leading to disease. Altogether, these findings might have implications for the pharmacological targeting of *Miro1* for the management and treatment of allergic airway diseases.

## Data Availability

The original contributions presented in the study are included in the article, further inquiries can be directed to the corresponding author.
